# Common Organisms and Their Drug Resistance Patterns in Endotracheal Aspirate Samples of Patients With Ventilator-Associated Pneumonia: A Retrospective Observational Study

**DOI:** 10.7759/cureus.115312

**Published:** 2026-08-27

**Authors:** Waqas Fazal, Kamal U Azam, Muhammad Ali, Shayan Aziz Khattak, Mohammad J Khan, Obaid Ullah, Zeeshan Khan, Suleman Khan, Lajward Khanum, Nabisha Zia

**Affiliations:** 1 Diabetes and Endocrinology, Royal Stoke University Hospital, Stoke-on-Trent, GBR; 2 Medicine, Hayatabad Medical Complex, Peshawar, PAK; 3 Internal Medicine, Salford Royal NHS Foundation Trust, Manchester, GBR; 4 Internal Medicine, Northwest General Hospital & Research Centre, Peshawar, PAK; 5 Medicine, Sunderland Royal Hospital, South Tyneside and Sunderland NHS Foundation Trust (STSFT), Sunderland, GBR; 6 Neurological Surgery, Northwest School of Medicine, Peshawar, PAK; 7 Internal Medicine, Khyber Teaching Hospital, Peshawar, PAK; 8 Internal Medicine, Gomal Medical College, Dera Ismail Khan, PAK

**Keywords:** acinetobacter, antibiotic susceptibility, intensive care unit, multidrug resistance, pseudomonas aeruginosa, ventilator-associated pneumonia

## Abstract

Background: Ventilator-associated pneumonia (VAP) is one of the important and serious nosocomial infections among critically ill patients, contributing to significant morbidity, mortality, and healthcare costs, particularly in resource-limited settings. Rising multidrug resistance (MDR) among causative organisms further complicates management and underscores the need for local antimicrobial surveillance.

Objective: This study aimed to determine the frequency of bacterial isolates and their antimicrobial susceptibility patterns in patients diagnosed with VAP in a tertiary care intensive care unit (ICU) in Peshawar, Pakistan.

Methods: A cross-sectional, retrospective observational study was conducted at the ICU of Northwest General Hospital from January to December 2024. Adult patients (≥18 years) who developed new-onset pneumonia-like symptoms at least 48 hours after endotracheal intubation were included. Endotracheal aspirate samples were cultured, and antimicrobial susceptibility testing was performed using the Kirby-Bauer disk diffusion method and interpreted according to Clinical and Laboratory Standards Institute (CLSI) M100, 34th edition (2024) guidelines. Data were analysed using IBM SPSS Statistics for Windows, Version 29 (Released 2022; IBM Corp., Armonk, New York) for descriptive and inferential statistics, with p < 0.05 considered statistically significant.

Results: Of 224 endotracheal aspirate samples analysed, 128 (57.1%) were from male patients, and the predominant age group was 60-74 years (67/224, 29.9%). The most frequently isolated organisms were *Acinetobacter *species (47/224, 21.0%; 95% CI: 16.2-26.8) and *Pseudomonas aeruginosa *(46/224, 20.5%; 95% CI: 15.8-26.3), followed by *Escherichia coli *(36/224, 16.1%; 95% CI: 11.8-21.4) and *Enterobacter* species (35/224, 15.6%; 95% CI: 11.5-21.0). High resistance rates were observed across several beta-lactam agents, particularly among *Acinetobacter*, *Enterobacter*, *Klebsiella*, and *Escherichia coli* isolates. Polymyxin demonstrated the highest overall in vitro susceptibility among the tested agents. Descriptive variation in organism distribution was observed across age groups; however, the association was not statistically significant (Pearson’s chi-square test of independence: χ²(8) = 7.98, p = 0.435).

Conclusion: *Acinetobacter *species and *Pseudomonas aeruginosa* were the most prevalent pathogens isolated from patients with VAP and showed high resistance across multiple antimicrobial classes. Polymyxin demonstrated the highest overall in vitro susceptibility and remains one of the most effective therapeutic options for these isolates, although treatment decisions should be guided by local antibiogram data, patient factors, and antimicrobial stewardship principles. These findings emphasise the urgent need for continuous local antibiogram monitoring and robust antibiotic stewardship programs to optimise empirical therapy and limit the spread of MDR organisms in ICU settings.

## Introduction

Ventilator-associated pneumonia (VAP) is defined as pneumonia in a patient on mechanical ventilation for >2 consecutive calendar days on the date of the event, with the day of ventilator placement as day 1, and the ventilator in place on the date of the event or the day before [1]. Ventilator-associated pneumonia has a reported incidence ranging from 5% to 40% among hospitalised ICU patients [2]. This wide range likely reflects differences in ICU populations, diagnostic criteria, surveillance methods, and duration of mechanical ventilation. The high incidence rate of VAP is concerning, as significant rates of mortality, morbidity, and increased hospitalisation present a burden on healthcare systems in low- and middle-income countries (LMICs) and low-income countries (LICs) [3,4]. Mortality rates range from 15% to 74%, with a mean hospital duration of 31.85 ± 18.96 days, and increasing costs of broad-spectrum antibiotics due to multidrug-resistant (MDR) strains (defined as non-susceptibility to at least one agent in three or more antimicrobial categories) impose a burden on both patients and healthcare providers [4,5]. Risk factors include prolonged mechanical ventilation, sedation, supine positioning, poor oral hygiene, muscle deconditioning, and re-intubation [6].

VAP is commonly categorised into early-onset and late-onset disease according to the timing of onset after initiation of mechanical ventilation. Early-onset VAP is often associated with organisms such as* Streptococcus pneumoniae*, *Haemophilus influenzae*, and sometimes *Staphylococcus aureus* (*S. aureus*), whereas late-onset VAP is more commonly associated with resistant Gram-negative organisms such as multidrug-resistant *Pseudomonas aeruginosa* (*P. aeruginosa*),* Acinetobacter spp.*, and methicillin-resistant *S. aureus* (MRSA) [7].

VAP is becoming increasingly challenging to treat due to the rising trend of antimicrobial resistance, leading to MDR pathogens, especially Gram-negative bacteria, which has resulted in increased morbidity and mortality, thus highlighting the need for antibiotic stewardship to mitigate adverse outcomes [8,9]. Despite being a common condition, it remains a relatively less researched topic in a resource-limited setting like Pakistan. The lack of local data to support the implementation of antibiotic stewardship or the adoption of updated guidelines for the management of VAP presents an obstacle. This retrospective observational study aims to identify patterns of bacterial pathogens and antimicrobial susceptibility profiles in endotracheal aspirate samples from patients with VAP to inform local antibiogram development and antimicrobial stewardship.

## Materials and methods

Study settings

The study was conducted at Northwest General Hospital (NWGH), a 650-bed tertiary care hospital in Peshawar, Pakistan, which receives a high volume of patients. The patients in this study were admitted to the Intensive Care Unit (ICU), which included a heterogeneous mix of medical and surgical patients suitable for assessing VAP and antimicrobial resistance patterns.

Study design and data collection

We conducted a cross-sectional, retrospective observational study from January 1, 2024, to December 31, 2024. Ethical approval was obtained from the Northwest General Hospital Institutional Review Board (IRB approval no. 7183). No formal sample size calculation was performed because this was a retrospective observational study. All eligible VAP cases during the study period with available endotracheal aspirate culture and antimicrobial susceptibility data were included consecutively using non-probability consecutive sampling, yielding a final sample size of 224 patients.

Ventilator-associated pneumonia was defined according to the Centers for Disease Control and Prevention (CDC)/National Healthcare Safety Network (NHSN) criteria as pneumonia occurring in a patient receiving invasive mechanical ventilation through an endotracheal or tracheostomy tube [1]. Cases were classified as VAP when the patient had been ventilated for more than two consecutive calendar days, with the day of ventilator initiation counted as day one, and the ventilator was in place on the date of the event or the preceding day. Eligible imaging evidence included a new and persistent or progressive and persistent infiltrate, consolidation, or cavitation on chest imaging. Clinically, the patient was required to have at least one systemic feature, including fever greater than 38.0°C, leukopenia of 4000 WBC/mm³ or less, or leucocytosis of 12,000 WBC/mm³ or more. Respiratory features included new onset or change in purulent sputum, increased respiratory secretions, worsening gas exchange such as oxygen desaturation, PaO₂/FiO₂ of 240 or less, increased oxygen requirement, or increased ventilator demand.

The samples for microbiological follow-up were collected by endotracheal aspiration using a 12-F suction catheter. Endotracheal aspirates were collected aseptically from mechanically ventilated patients through the artificial airway and processed after confirmation that the sample was representative of lower respiratory tract secretions. Samples were cultured on blood agar and MacConkey agar, and significant bacterial growth was defined as >10^5^ CFU/mL. Bacterial isolates were identified using standard bacteriological methods. Antimicrobial susceptibility testing (AST) was performed using the Kirby-Bauer disk diffusion method on Mueller-Hinton agar and interpreted according to the Clinical and Laboratory Standards Institute (CLSI) M100, 34th edition (2024) guidelines. Polymyxin susceptibility testing was performed using broth microdilution and interpreted according to European Committee on Antimicrobial Susceptibility Testing (EUCAST) guidelines. Where clear breakpoints were not available, historical breakpoints or FDA breakpoints were used. Where validated breakpoints were not available, minimum inhibitory concentration (MIC) values were reported by the broth microdilution method and interpreted using the EUCAST breakpoints (version 11.0). Routine laboratory quality-control procedures were followed in accordance with established microbiology laboratory protocols and CLSI recommendations.

All adult patients above the age of 18 years who developed new-onset pulmonary symptoms at least 48 hours after intubation for mechanical ventilation were deemed eligible for the study. Exclusion criteria included age less than 18 years, pre-existing pulmonary infectious conditions, or pneumonia on admission or during the first 72 hours of mechanical ventilation. Duplicate data points were manually screened for and removed.

Patients’ demographics and admission details were obtained from an existing patient database. Ventilation initiation and clinical notes regarding the symptoms were extracted from the Hospital Management Information System (HMIS). Furthermore, microbiological culture results and antibiotic susceptibility reports were obtained from the Laboratory Information System (LIS), and data extraction was performed without direct patient contact.

Statistical analysis

Descriptive statistics were used to summarise demographic, microbiological, and antimicrobial susceptibility data. Categorical variables are presented as numbers and percentages (n (%)). For major pathogen frequencies, 95% confidence intervals were calculated using the Wilson score method. For antimicrobial susceptibility testing, percentages were calculated using the number of isolates tested for each organism-antibiotic combination as the denominator. Associations between age group and organism category were assessed using Pearson’s chi-square test of independence. Chi-square statistics, degrees of freedom, and two-sided p-values are reported, with p < 0.05 considered statistically significant.

## Results

A total of 224 endotracheal aspirate samples were analysed for bacterial growth and antimicrobial susceptibility patterns.

The age distribution of patients is summarised in Table 1. The majority of patients were aged 60-74 years, comprising 67/224 (29.9%) of the cohort. This was followed by patients aged 45-59 years, with 56/224 (25.0%), and those aged 30-44 years, with 42/224 (18.8%). Patients aged ≥75 years accounted for 38/224 (17.0%), whereas those aged 18-29 years were the least represented group, comprising 21/224 (9.4%).

**Table 1:** Age distribution of patients

Age	Frequency	Percentage
18-29	21	9.4
30-44	42	18.8
45-59	56	25.0
60-74	67	29.9
75+	38	17.0

**Figure 1:**
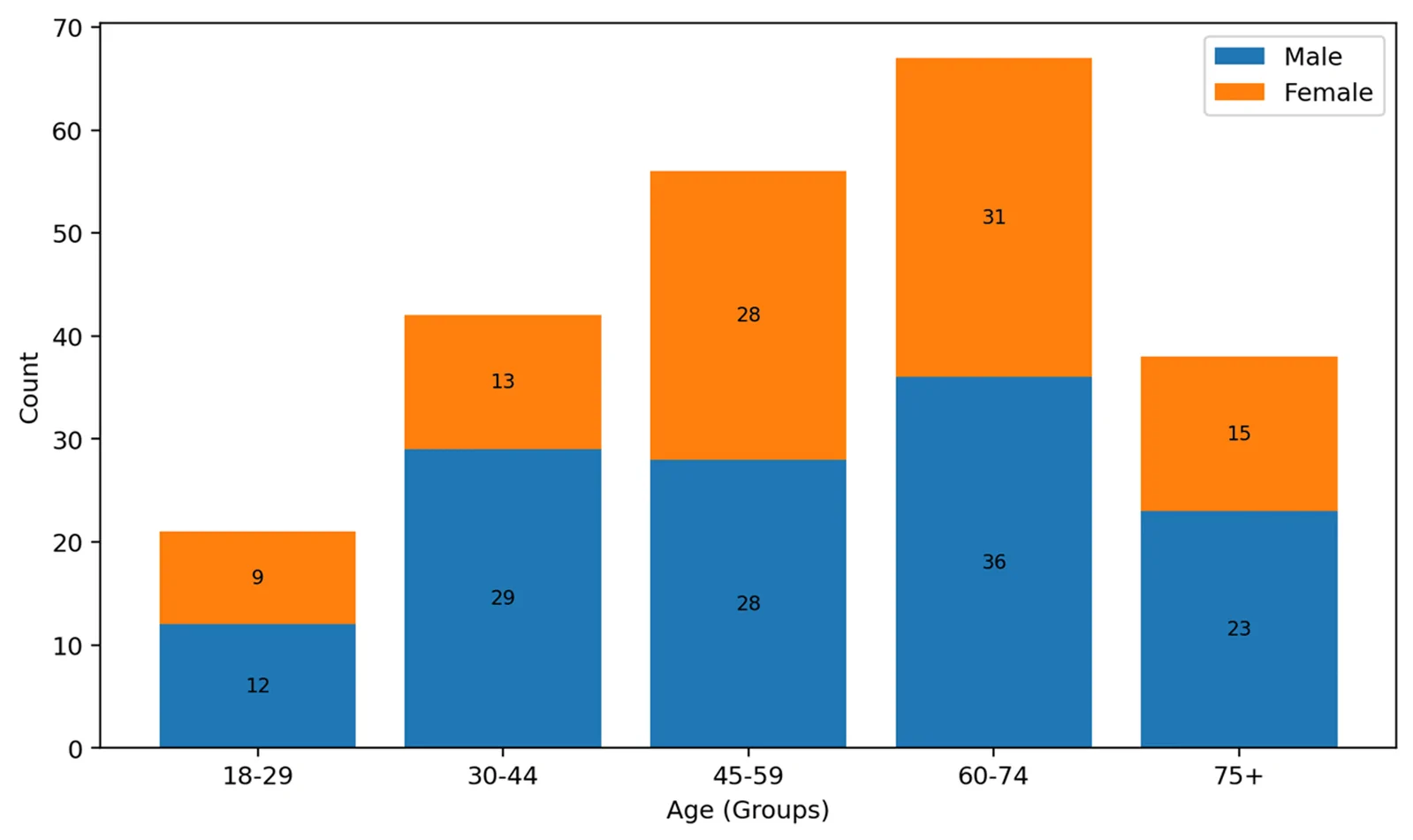
Sex distribution across age groups

Figure 1 shows the distribution of male and female patients across age groups. Overall, there was a male predominance, with 128/224 males (57.1%) and 96/224 females (42.9%), yielding a male-to-female ratio of approximately 1.33:1.

The frequency of bacterial isolates is detailed in Table 2. The most frequently identified organism was *Acinetobacter* species (47/224, 21.0%; 95% CI: 16.2-26.8), followed by *Pseudomonas aeruginosa* (46/224, 20.5%; 95% CI: 15.8-26.3), *Escherichia coli* (36/224, 16.1%; 95% CI: 11.8-21.4), and *Enterobacter* species (35/224, 15.6%; 95% CI: 11.5-21.0). Less commonly isolated pathogens included *Klebsiella* species, non-aeruginosa *Pseudomonas* species, *coliform* species, *Providencia* species, *Proteus mirabilis*, *Streptococcus species*, *Citrobacter* species, and *Serratia* species. Rare isolates included *Staphylococcus aureus* (2/224, 0.9%), *Burkholderia cepacia* (1/224, 0.4%), and *Proteus vulgaris* (1/224, 0.4%). In Table 2, “*Pseudomonas* species” refers to non-aeruginosa *Pseudomonas* isolates and is reported separately from *Pseudomonas aeruginosa.*

**Table 2:** Frequency of isolated organisms Coliform species refers to isolates reported by the microbiology laboratory as coliforms without further speciation.

Organism	Frequency	Percentage
Acinetobacter species	47	21.0
Pseudomonas aeruginosa	46	20.5
Escherichia coli	36	16.1
Enterobacter species	35	15.6
Klebsiella species	17	7.6
Non-aeruginosa *Pseudomonas species*	15	6.7
Coliform species	5	2.2
Providencia species	5	2.2
Proteus mirabilis	4	1.8
Streptococcus species	4	1.8
Citrobacter species	3	1.3
Serratia species	3	1.3
Staphylococcus aureus	2	0.9
Burkholderia cepacia	1	0.4
Proteus vulgaris	1	0.4
Total	224	100

Antimicrobial susceptibility patterns of the six most frequently isolated organisms are presented in Table 3. Values are reported as n/N (%), where N represents the number of isolates tested for each organism-antibiotic combination.

**Table 3:** Antimicrobial susceptibility patterns of the six most frequent organisms, shown as n/N (%) R: resistant; S: susceptible. Values are shown as n/N (%), where N represents the number of isolates tested for each organism-antibiotic combination. Percentages were calculated as R/(R+S) × 100 for resistance and S/(R+S) × 100 for susceptibility.

Antibiotic	Result	Acinetobacter spp.	Pseudomonas aeruginosa	Escherichia coli	Enterobacter spp.	Klebsiella spp.	Non-aeruginosa Pseudomonas spp.
Amikacin	R	28/37 (75.7%)	17/37 (45.9%)	8/31 (25.8%)	22/32 (68.8%)	12/16 (75.0%)	8/15 (53.3%)
Amikacin	S	9/37 (24.3%)	20/37 (54.1%)	23/31 (74.2%)	10/32 (31.2%)	4/16 (25.0%)	7/15 (46.7%)
Cefixime	R	11/12 (91.7%)	18/19 (94.7%)	7/8 (87.5%)	13/13 (100.0%)	1/1 (100.0%)	2/2 (100.0%)
Cefixime	S	1/12 (8.3%)	1/19 (5.3%)	1/8 (12.5%)	0/13 (0.0%)	0/1 (0.0%)	0/2 (0.0%)
Cefoperazone	R	30/31 (96.8%)	11/21 (52.4%)	25/27 (92.6%)	21/21 (100.0%)	11/11 (100.0%)	6/10 (60.0%)
Cefoperazone	S	1/31 (3.2%)	10/21 (47.6%)	2/27 (7.4%)	0/21 (0.0%)	0/11 (0.0%)	4/10 (40.0%)
Cefotaxime	R	16/17 (94.1%)	11/12 (91.7%)	16/17 (94.1%)	16/16 (100.0%)	8/8 (100.0%)	4/5 (80.0%)
Cefotaxime	S	1/17 (5.9%)	1/12 (8.3%)	1/17 (5.9%)	0/16 (0.0%)	0/8 (0.0%)	1/5 (20.0%)
Ceftazidime	R	32/32 (100.0%)	18/31 (58.1%)	28/29 (96.6%)	22/22 (100.0%)	16/16 (100.0%)	5/10 (50.0%)
Ceftazidime	S	0/32 (0.0%)	13/31 (41.9%)	1/29 (3.4%)	0/22 (0.0%)	0/16 (0.0%)	5/10 (50.0%)
Ceftriaxone	R	22/23 (95.7%)	20/25 (80.0%)	17/17 (100.0%)	13/13 (100.0%)	8/8 (100.0%)	7/8 (87.5%)
Ceftriaxone	S	1/23 (4.3%)	5/25 (20.0%)	0/17 (0.0%)	0/13 (0.0%)	0/8 (0.0%)	1/8 (12.5%)
Ciprofloxacin	R	25/25 (100.0%)	5/16 (31.2%)	22/24 (91.7%)	11/12 (91.7%)	11/11 (100.0%)	3/8 (37.5%)
Ciprofloxacin	S	0/25 (0.0%)	11/16 (68.8%)	2/24 (8.3%)	1/12 (8.3%)	0/11 (0.0%)	5/8 (62.5%)
Clindamycin	R	21/22 (95.5%)	20/21 (95.2%)	23/23 (100.0%)	14/14 (100.0%)	6/6 (100.0%)	5/5 (100.0%)
Clindamycin	S	1/22 (4.5%)	1/21 (4.8%)	0/23 (0.0%)	0/14 (0.0%)	0/6 (0.0%)	0/5 (0.0%)
Co-amoxiclav	R	13/13 (100.0%)	21/21 (100.0%)	18/19 (94.7%)	14/14 (100.0%)	9/9 (100.0%)	8/8 (100.0%)
Co-amoxiclav	S	0/13 (0.0%)	0/21 (0.0%)	1/19 (5.3%)	0/14 (0.0%)	0/9 (0.0%)	0/8 (0.0%)
Gentamicin	R	18/26 (69.2%)	13/23 (56.5%)	12/27 (44.4%)	13/17 (76.5%)	9/12 (75.0%)	7/12 (58.3%)
Gentamicin	S	8/26 (30.8%)	10/23 (43.5%)	15/27 (55.6%)	4/17 (23.5%)	3/12 (25.0%)	5/12 (41.7%)
Imipenem	R	18/20 (90.0%)	15/25 (60.0%)	5/18 (27.8%)	14/18 (77.8%)	8/13 (61.5%)	6/13 (46.2%)
Imipenem	S	2/20 (10.0%)	10/25 (40.0%)	13/18 (72.2%)	4/18 (22.2%)	5/13 (38.5%)	7/13 (53.8%)
Meropenem	R	28/28 (100.0%)	11/22 (50.0%)	11/20 (55.0%)	16/19 (84.2%)	6/7 (85.7%)	3/5 (60.0%)
Meropenem	S	0/28 (0.0%)	11/22 (50.0%)	9/20 (45.0%)	3/19 (15.8%)	1/7 (14.3%)	2/5 (40.0%)
Piperacillin	R	37/37 (100.0%)	17/33 (51.5%)	27/28 (96.4%)	24/24 (100.0%)	16/16 (100.0%)	7/11 (63.6%)
Piperacillin	S	0/37 (0.0%)	16/33 (48.5%)	1/28 (3.6%)	0/24 (0.0%)	0/16 (0.0%)	4/11 (36.4%)
Polymyxin	R	3/33 (9.1%)	3/29 (10.3%)	3/29 (10.3%)	5/23 (21.7%)	0/16 (0.0%)	0/11 (0.0%)
Polymyxin	S	30/33 (90.9%)	26/29 (89.7%)	26/29 (89.7%)	18/23 (78.3%)	16/16 (100.0%)	11/11 (100.0%)
Tigecycline	R	1/19 (5.3%)	18/19 (94.7%)	1/28 (3.6%)	1/17 (5.9%)	0/8 (0.0%)	7/9 (77.8%)
Tigecycline	S	18/19 (94.7%)	1/19 (5.3%)	27/28 (96.4%)	16/17 (94.1%)	8/8 (100.0%)	2/9 (22.2%)
Cefepime	R	26/26 (100.0%)	13/22 (59.1%)	26/27 (96.3%)	16/16 (100.0%)	11/11 (100.0%)	3/7 (42.9%)
Cefepime	S	0/26 (0.0%)	9/22 (40.9%)	1/27 (3.7%)	0/16 (0.0%)	0/11 (0.0%)	4/7 (57.1%)

Overall, high resistance was observed across several β-lactam agents, particularly among *Acinetobacter* species, *Enterobacter* species, *Klebsiella* species, and *Escherichia coli*. All tested isolates of *Acinetobacter* species showed resistance to meropenem, ceftazidime, cefepime, ciprofloxacin, piperacillin, and co-amoxiclav. All tested isolates of *Enterobacter* species and *Klebsiella* species also demonstrated resistance to multiple cephalosporins and β-lactam agents. *Escherichia coli* showed high resistance to ceftriaxone, ceftazidime, piperacillin, cefepime, co-amoxiclav, cefotaxime, cefoperazone, and ciprofloxacin.

*Pseudomonas aeruginosa* showed resistance to co-amoxiclav in all tested isolates and high resistance to cefixime, tigecycline, cefotaxime, and ceftriaxone. Non-aeruginosa *Pseudomonas* species showed resistance in all tested isolates to co-amoxiclav and cefixime, with variable resistance to other agents.

Polymyxin demonstrated the highest overall in vitro susceptibility among the tested agents. Susceptibility to polymyxin was retained in 30/33 *Acinetobacter* isolates (90.9%), 26/29 *Pseudomonas aeruginosa* isolates (89.7%), 26/29 *Escherichia coli* isolates (89.7%), 18/23 *Enterobacter* isolates (78.3%), 16/16 *Klebsiella* isolates (100.0%), and 11/11 non-aeruginosa *Pseudomonas* isolates (100.0%). Tigecycline also showed high in vitro susceptibility among *Acinetobacter*, *Escherichia coli*, *Enterobacter*, and *Klebsiella* isolates, but resistance was high among *Pseudomonas aeruginosa* and non-aeruginosa *Pseudomonas* species.

**Figure 2:**
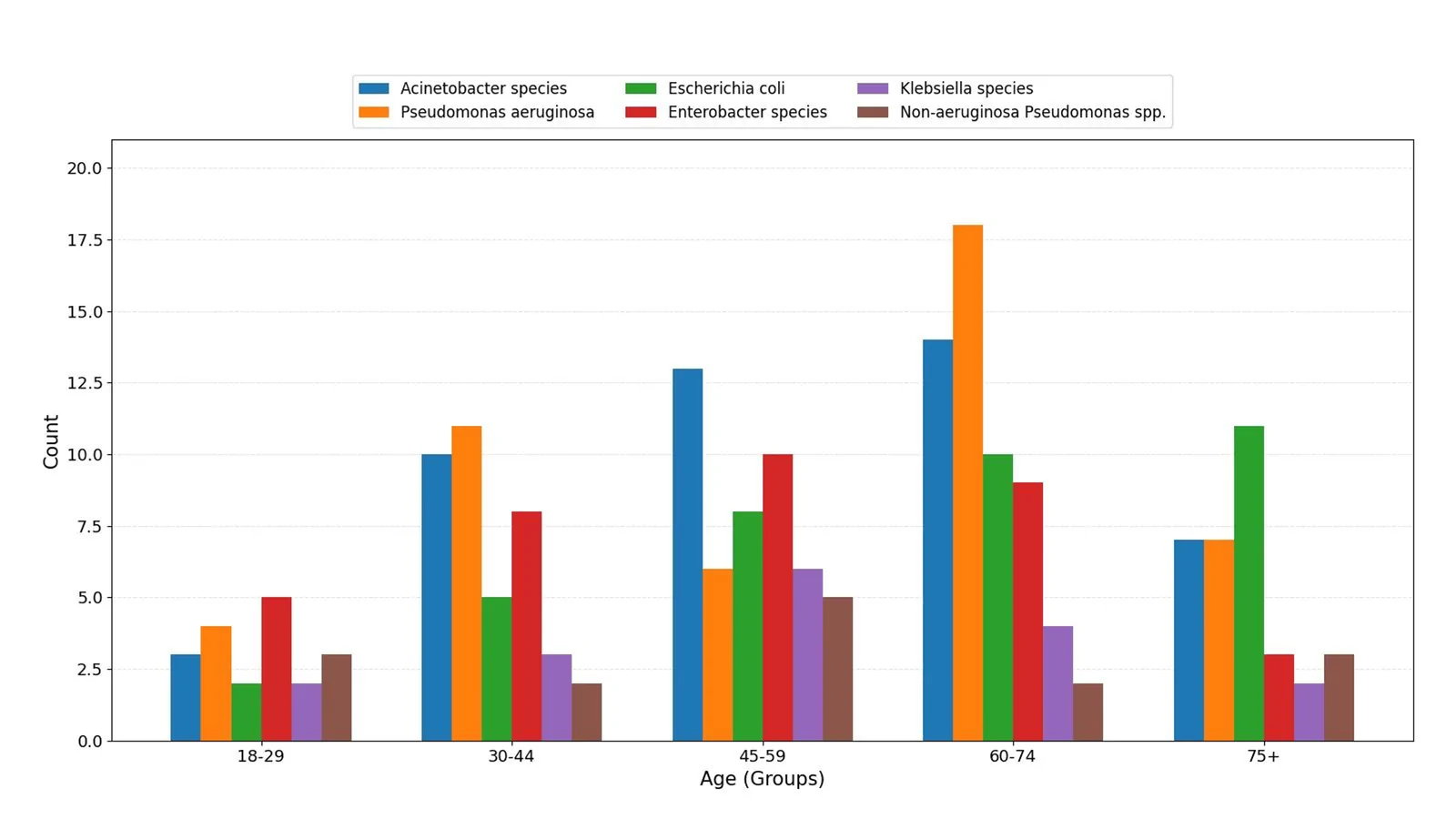
Age distribution of the six most frequently isolated organisms Differences in the distribution of the three most frequent organisms across age groups (Pearson’s chi-square test of independence: χ²(8) = 7.98, p = 0.435).

A comparative analysis of bacterial prevalence across age groups is shown in Figure 2. Descriptively, *Acinetobacter* species and *Pseudomonas aeruginosa* were most frequently observed in the 60-74-year age group, with 14 and 18 isolates, respectively. *Escherichia coli* was most frequent in the ≥75-year age group, with 11 isolates, whereas *Enterobacter* species was most frequent in the 45- to 59-year age group, with 10 isolates. However, these descriptive differences should be interpreted cautiously because the association between age group and organism distribution was not statistically significant.

A Pearson chi-square test of independence was used to assess the association between age group and organism distribution. When the three most frequently isolated organisms, i.e., *Acinetobacter* species, *Pseudomonas aeruginosa*, and *Escherichia coli, *were compared across age groups, no statistically significant association was observed (χ²(8) = 7.98, p = 0.435).

## Discussion

The main objective of this study was to identify the microbiological growth patterns in endotracheal aspirate samples from patients who developed VAP. We stratified our population into age and sex subgroups and then assessed microbiological growth and antibiotic resistance patterns in endotracheal aspirate samples. The primary aim was to create a foundation for antibacterial stewardship that better reflects our local clinical setup. As previous studies have noted, this process of quantifying the etiological agents for VAP in individual setups is paramount to the accurate treatment of VAP [10-13]

Looking at age variations, few studies have stratified VAP patient populations into individual age brackets, but determining the mean age is common. In our study, the most common age group was 60-74 years old, with a unimodal distribution. This compares with the findings of many other studies such as those by Chastre et al. [14], in which the mean age was 61 years (SD ±13), Markowicz et al. [15], in which the mean was 54 years (SD ±15), Trouillet et al. [16], in which the mean was 62.8 years old (SD ±13.2), Rello et al. [17], in which the mean age was 64.1 years (SD ±13.1), and Chang et al. [18], in which the mean age was 69 years (59-76). This underlines the concept of age being a predominant risk factor for developing VAP, with the overall risk being highest in the ages above 60 years [10,14-17].

In contrast, Goel et al. [19], in their prospective study of ICU-admitted patients, reported an average age of 46.4 years (SD ±18.45). This study was conducted in India and would have environmental and population demographics similar to our own, but the difference in average age is notable. At the other extreme, Unver et al. [20], in their retrospective study, found an average age of 71.4 years, much higher than in other studies. Such findings were replicated in Kepekci et al.'s retrospective study [21], in which the average age was 72.05 years (SD ±14.18); both studies, conducted in tertiary care hospitals in Turkey, showed results that contrast with our study.

A study with similar location and design was conducted by Wajahat et al. [22]. In their study, they stratified ages into age brackets and found a unimodal distribution, with the peak age frequency in the 31-60-year-old bracket. Similarly, Farag et al. [23] stratified by age and found a bimodal distribution with two peaks at the 31-45-year-old and >60-year-old brackets. These age brackets show a much younger mean age than in our study. 

Our study also showed an overall male predominance of VAP, with 128/224 patients (57.1%) being male. Previous studies have similarly reported a predominance of male patients with VAP [14-17], with some studies reporting male proportions ranging from 60% to 80%. Male predominance has also been reported in settings similar to ours [19,22,24]. The reasons for this association remain uncertain and may reflect differences in patient demographics, comorbidities, healthcare exposure, or other unmeasured factors. 

Moving to the primary focus of our study, Chastre* *et al. and Fagon et al. reported in their review that* Pseudomonas aeruginosa* (24.4%), *Staphylococcus aureus* (20.4%), and *Enterobacteriaceae* (14.1%) were among the most commonly isolated organisms in VAP [10]. In comparison,* Acinetobacter* species was the most common organism in our study, identified in 47/224 samples (21.0%), followed by *Pseudomonas aeruginosa* in 46/224 samples (20.5%). *Staphylococcus aureus* was rare in our study, identified in 2/224 samples (0.9%). *Enterobacter* species were isolated in 35/224 samples (15.6%); however, this should not be interpreted as equivalent to the broader *Enterobacteriaceae* group reported by Chastre et al.* *and Fagon* *et al. Overall, Gram-negative organisms predominated in our cohort, particularly *Acinetobacter* species,* Pseudomonas aeruginosa, Escherichia coli, *and* Enterobacter species.*

Other findings by Chastre et al. [10] were that Gram-positive organisms were a leading cause of early-onset VAP, whereas Gram-negative organisms were usually associated with late VAP (>5-7 days). They also reported that antimicrobial resistance is a rising issue, with *Pseudomonas*, *Enterobacteriaceae,* and *Acinetobacter* becoming resistant to piperacillin, aztreonam, and early-generation cephalosporins due to the presence of Class I cephalosporinases and extended-spectrum β-lactamases. This issue of resistance to carbapenems and cephalosporins is highlighted in our study as well, with *Acinetobacter, Pseudomonas, E. coli,* and *Enterobacter* being almost fully resistant to Β-lactam antibiotics and fluoroquinolones, with some retained susceptibility to amikacin, tigecycline, and polymyxin. This may challenge the findings of the Chastre et al. study, which recommends using Β-lactam antibiotics such as antipseudomonal penicillins and combinations of second- and third-generation cephalosporins with Β-Lactam- β-Lactamase inhibitors. 

Further studies by Fagon et al. [11], Kalanuria et al. [12], Chastre et al. [14], Markowicz et al*.* [15], and Trouillet et al. [16] all report similar results that contrast with our own. They too report that in the early VAP, Gram-positive aerobic organisms such as *Streptococcus, Staphylococcus, *and* Haemophilus influenzae *predominate, and in late VAP, the cultures are dominated by multidrug-resistant* Pseudomonas, Acinetobacter, *and* Enterobacteriaceae*. However, as in our study, in situations where stratification into early and late VAP was not performed [14,15], the overall trends matched our findings, with *Pseudomonas* as the most common isolate, followed by Gram-positive cocci and then *Acinetobacter*. One thing to note is that the time cut-off for late VAP is ill-defined across these studies and varies from 4 to 7 days.

This distinction between early and late VAP is important, and the choice of antibacterial therapy changes based on duration of ventilation. Many studies [10,11,16,17] state that therapy with penicillin-class antibiotics, combined with a β-lactamase inhibitor, would be adequate for early VAP, as this is mainly attributed to Gram-positive cocci that display good sensitivity profiles to β-lactam antibiotics, such as amoxicillin or second-generation cephalosporin. Combination therapy with carbapenem, aminoglycoside/fluoroquinolone, and vancomycin would be reserved for late VAP, where the pathogen grown is more resistant. To challenge this, our study shows that the proportion of multidrug-resistant Gram-negative organisms is much higher, and perhaps the early use of broad-spectrum antibiotics would be something to consider, such as an antipseudomonal cephalosporin or an antipseudomonal penicillin with a β-lactamase inhibitor in addition to aminoglycosides or ciprofloxacin [16]. This hypothesis has not been fully investigated and would be an important consideration for future research, especially in the context of rising drug resistance patterns stimulated by inappropriate antibiotic stewardship. However, we stress the need to follow local antibiotic guidelines and protocols in such situations. 

Comparing our results with studies in which the population demographics more closely matched ours, we note the findings of Noyal et al. [13]. In their prospective observational study they noted the primary pathogen in early VAP were* Enterobacteriaceae* (25%) and *Acinetobacter* (25%), followed by *Staphylococcus aureus *(13%). In late VAP, *Pseudomonas* (39%) and *Acinetobacter* (32%) were common. This finding of *Acinetobacter* being common early on and *Pseudomonas* being common overall is congruent with our findings. They also noted a high prevalence of multidrug resistance such as extended-spectrum-β-lactamase, Amp-C β-lactamase, and metallo-β-lactamases. They reported nearly full resistance to teicoplanin, gentamicin, ciprofloxacin and meropenem, especially in *Acinetobacter, E. coli, and Pseudomonas*. This mirrors our sensitivity profiles very closely. Interestingly, in isolates grown from late VAP, the species of *Acinetobacter *and *Pseudomonas* showed higher sensitivity to teicoplanin, piperacillin and meropenem. Our study did not differentiate between early and late cultures of the same pathogen.

Bhaskar et al. [25] reported findings that differed from those of the previously discussed studies. Their most common isolates were *Citrobacter* (52.83%) and *Klebsiella* species (13.21%). Among these Gram-negative isolates, high resistance was observed to gentamicin (82%), amikacin (82%), cephalosporins (90%), and piperacillin (72%), whereas susceptibility was reported to tigecycline (96%), imipenem (50%), and polymyxin (96%). This resistance pattern resembles ours, with high resistance to β-lactam antibiotics and greater susceptibility to polymyxin and tigecycline. However, the predominance of *Citrobacter* was distinctive to their study. Other studies, including those by Ergület al. [26], Chang et al. [18], and Goel et al*.* [19], have similarly reported Gram-negative organisms such as *Acinetobacter, Pseudomonas, *and *Klebsiella* species as common pathogens, with variable susceptibility to piperacillin, amikacin, and cephalosporins.

Looking closer at studies done in similar geographical areas, Wajahat et al. [22] in their study found *Acinetobacter *the most common (36.11%) pathogen with increased susceptibility to carbapenems (95.65%) and piperacillin (90.48%) but near full resistance to polymyxin, the second most common isolate was *Pseudomonas *(13.89), which exhibited similar sensitivities to carbapenems (40-100%) but much better sensitivity to polymyxin (77.76%) and much lower sensitivity to piperacillin (50%). This is similar to our results in terms of the bacterial type isolated, but different in terms of sensitivity patterns to β-Lactams and other antibiotics. It is in relation to these variable resistance patterns that Wajahat et al. noted the value in combination therapy needed earlier in setups with high β-lactam resistance rates.

A consistent finding with all these studies was that β-lactam resistance was exceedingly common in Gram-negative pathogens, which unfortunately were also usually the most common pathogens grown in VAP in the ICU setups of the developing world. Perhaps the majority of these studies can be divided into two subcategories: ones where gram-negatives were the primary pathogen, such as *Acinetobacter, Pseudomonas, Klebsiella,* and* Proteus*, presenting with resistance to higher-end antibiotics; and the other being those where Gram-positive cocci were more common early, with susceptibility patterns throughout and gram-negative later, with high resistance patterns. The division perhaps splitting the first category into low- and middle-income countries and the latter into high-income countries. Relating these pattern differences to external factors such as healthcare delivery, antibiotic stewardship, and ICU care would be an interesting study.

Our final result is of growth pattern stratified into age categories; this is a finding very few studies have investigated and is difficult to relate to the bigger picture. Our finding of resistant gram-negative organisms such as *Acinetobacter *and *Pseudomonas *being overrepresented in older age groups is an interesting finding and may relate to inherent immunological and epigenetic changes. This growth variability may be reflected in the meta-analysis by Aliyu et al. [27], where they looked at community-acquired pneumonia growth patterns in nursing home residents and found high rates of Gram-negative organisms. Although this population is far removed from our own, they noted that this unique finding warrants further research into why it occurs. However, statistical analysis showed that this association was not significant. Further studies with larger sample sizes may be valuable to explore this relationship in greater detail.

In conclusion, our study had limitations. It was a retrospective study conducted at a single tertiary care centre, which may limit generalisability and introduce selection bias. The study did not include clinical outcome data such as ICU mortality, hospital mortality, ICU length of stay, hospital length of stay, patient survival, or duration of mechanical ventilation. Data on prior antibiotic exposure, comorbidities, severity of illness, and other potential risk factors for multidrug-resistant organisms were also not analysed. Formal organism-specific MDR prevalence was not calculated because isolate-level resistance classification across antimicrobial categories was not available for all isolates. In addition, the study did not subdivide VAP into early- and late-onset categories, limiting direct comparison with studies that stratify cases by timing of onset. Endotracheal aspirate cultures are less invasive and more feasible than bronchoalveolar lavage or protected specimen brush sampling, but they may be more vulnerable to contamination or colonisation and therefore must be interpreted alongside clinical and radiological criteria. Because of the retrospective design, we were unable to determine which clinical factors influenced the distribution of organisms or resistance patterns. Multicentre prospective studies incorporating clinical outcomes, prior antibiotic exposure, duration of ventilation, and MDR risk-factor analysis would provide more robust evidence.

## Conclusions

This study provides local microbiological surveillance data on bacterial isolates and antimicrobial susceptibility patterns among patients with VAP in a tertiary care ICU. *Acinetobacter* species and *Pseudomonas aeruginosa* were the most frequently isolated organisms, and several Gram-negative isolates showed high resistance to multiple antimicrobial classes, including carbapenems and cephalosporins. Polymyxin demonstrated the highest overall in vitro susceptibility among the tested agents and remains one of the most effective therapeutic options for these isolates, although clinical use should be guided by patient factors, local susceptibility results, and antimicrobial stewardship principles.

These findings support the need for regular institutional antibiogram monitoring, robust infection-control practices, and antimicrobial stewardship programmes. Because this was a retrospective microbiological study without clinical outcome analysis, the results should be interpreted as local surveillance data rather than direct evidence for changes in empirical treatment strategies. Prospective studies incorporating clinical outcomes, prior antibiotic exposure, duration of ventilation, and MDR risk-factor analysis are needed to better guide treatment decisions.
